# Geographic Variability of Biologically Active Compounds, Antioxidant Activity and Physico-Chemical Properties in Wild Bilberries (*Vaccinium myrtillus* L.)

**DOI:** 10.3390/antiox11030588

**Published:** 2022-03-19

**Authors:** Dalia Urbonaviciene, Ramune Bobinaite, Pranas Viskelis, Ceslovas Bobinas, Aistis Petruskevicius, Linards Klavins, Jonas Viskelis

**Affiliations:** 1 Lithuanian Research Centre for Agriculture and Forestry, Institute of Horticulture, 54333 Babtai, Lithuania; ramune.bobinaite@gmail.com (R.B.); pranas.viskelis@lammc.lt (P.V.); ceslovas.bobinas@lammc.lt (C.B.); aistis.petruskevicius@lammc.lt (A.P.); jonas.viskelis@lammc.lt (J.V.); 2 Department of Environmental Science, University of Latvia, 1004 Riga, Latvia; linards.klavins@lu.lv

**Keywords:** bilberry, natural habitats, polyphenol, anthocyanins, antioxidant activity, different geographical locations

## Abstract

The aim of this study was to characterize the variation in biologically active compounds, antioxidant activity and physico-chemical properties in naturally grown bilberries gathered from different sites in Northern Europe. The variability in the biologically active compounds, antioxidant capacity and physico-chemical properties, as well as the development of tools for the authenticity and quality control of wild bilberries (*V. myrtillus* L.) in different geographical locations was evaluated. The berries of bilberries were handpicked during the summers of 2019 and 2020 during the time periods when they are typically harvested for commercial purposes in Northern Europe (Norway (NOR), Finland (FIN), Latvia (LVA) and Lithuania (LTU)). Berries from locations in NOR were distinguished by their higher mean TPC (791 mg/100 g FW, average), whereas the mean TPC of samples from the most southern country, LTU, was the lowest (587 mg/100 g FW). The TPC of bilberries ranged from 452 to 902 mg/100 g FW. The TAC values of investigated bilberry samples varied from 233 to 476 mg/100 g FW. A high positive correlation was found between TPC and antioxidant activity of the bilberry samples (R = 0.88 and 0.91 (FRAP and ABTS assays, respectively)), whereas the correlation between TAC and antioxidant activity was lower (R = 0.65 and 0.60). There were variations in the TPC and TAC values of investigated berries, suggesting that genotype also affects the TPC and TAC in berries. In 2020, the pH values and TSS contents of berries were significantly lower than in 2019. To the best of our knowledge, this is the first comprehensive reported evaluation of the biologically active compounds in wild bilberries from different Northern European countries using one laboratory-validated method.

## 1. Introduction

Authenticity and traceability are very important criterions of product quality. In order to assure quality, the chemometric approach can be efficiently used for berries and their products. Phenolic compounds are the major group of phytochemicals found in berries. They possess one or more aromatic rings with hydroxyl groups and their structures may range from that of a simple phenolic molecule to that of a complex high-molecular mass polymer [1]. The content of phenolic compounds in berries is determined by many factors, such as genotype, growing conditions, ripeness, storage time and other conditions [2,3,4,5]. Researchers have shown that the fruits of berry plants that grow in a cold climate with a short vegetation season and without fertilizers are marked by higher contents of phenolic compounds than the same varieties that grow in a milder climate [6,7]. Phenolic compounds can be used for the assessment of authenticity since genotype has a profound impact on the concentration and qualitative composition of these compounds in berries [2,8,9].

Anthocyanin pigments represent a very important group of phenolic substances because they determine the color of both berry fruits and their products [10]. Anthocyanins are glycosides of anthocyanidins of which the most important in the plants are: cyanidin, delphinidin, pelargonidin, peonidin, malvidin and petunidin. The distribution of these anthocyanidins in fruits and vegetables is 50%, 12%, 12%, 12%, 7% and 7%, respectively [11]. Glucose and rhamnose are the more common sugar moieties attached to aglycone (anthocyanidin), but galactose, arabinose, xylose, rutinose, sambubiose and other sugars are also frequently found [12]. Anthocyanins are usually present in colored flavylium cation form but may also be in uncolored form depending on the pH. This characteristic is an important issue when analyzing anthocyanins [13].

The berry genotype predetermines its typical profile of anthocyanins [10,14], thus the anthocyanin profile can be used to identify berry variety [10] for product quality control, label claim verification and raw material source identification [15].

The official method of anthocyanin pigment analysis is the spectrophotometric pH differential method. It is a rapid and simple quantitative method. The pH differential method is not useful for qualitative analysis of anthocyanins [16], but on the other hand, the spectrophotometric method for the quantification of total anthocyanins is widely used for the standardization of natural raw materials [17]. Other validated methods for identification and quantification of anthocyanins include high-pressure liquid chromatography (HPLC) [18,19]. In general, liquid chromatography remains the most common technique used for identification and characterization of anthocyanins in fruit extracts, whereas its combination with electrospray ionization mass spectrometry (ESI-MS) [15] or quadrupole/time-of-flight mass spectrometry (QTOF-MS) gives better quantitation of anthocyanins in more complex samples [19,20], because retention times, UV–vis spectra, mass/charge ratios as well as the type of anthocyanidin and the nature of sugar substitution can be easily recorded and used together to solve anthocyanin structures [21].

Different laboratories use different analysis parameters and conditions in order to optimize separation, thus it is difficult to point to one single (standard) HPLC procedure for determination of anthocyanins [19], but the columns chosen for the determination of anthocyanins are almost exclusively reverse phase columns composed of a C18 stationary phase. The solvent system (mobile phase) typically consists of an aqueous phase and an organic phase (mainly methanol or acetonitrile) with the acidic modifier (mainly formic or acetic acid) [19,22]. Acidic modifiers ensure that anthocyanins predominantly exist in their colored (flavylium cationic) form, having a maximum absorbance in the visible region around 520 nm [23].

The acid hydrolysis HPLC method simplifies the anthocyanin profile of berry samples by converting anthocyanins to anthocyanidin aglycones that can be separated and quantified using external standards. However, in this case part of the information concerning the precise composition and quantity of individual anthocyanins present is lost [24,25]. Furthermore, acid hydrolysis as part of the sample preparation tends to overestimate the anthocyanin content, since proanthocyanidins may be de-polymerized and converted into anthocyanidins [26].

Bilberry (*Vaccinium myrtillus* L.) is one of the richest sources of anthocyanin pigments among berry fruits [27,28]. These colored compounds accumulate not only in the skin of bilberries but also throughout the fruit flesh [25]. Bilberries are characterized by 15 anthocyanins. These include delphinidin, cyanidin, petunidin, peonidin and malvidin, glycosidically linked to glucose, arabinose or galactose [24,27,29]. As mentioned previously, the HPLC profiles of anthocyanins and anthocyanidins present in berries can be used as fingerprints for the evaluation of the authenticity of raw materials [30] as well as finished food products [31].

The aim of this study was to characterize the variation in biologically active compounds, antioxidant activity and physico-chemical properties in naturally grown bilberries gathered from different sites in Northern Europe. The variability of the biologically active compounds (polyphenol (TPC) and total anthocyanin (TAC) content), antioxidant capacity (FRAP; ABTS RSA) and physico-chemical properties (total soluble solids (TSS), pH), as well as the development of tools for the authenticity and quality control of wild bilberries (*V. myrtillus* L.) in different geographical locations was evaluated.

## 2. Materials and Methods

### 2.1. Extraction

For determination of anthocyanins, total phenols and antioxidant activity in 50 g of defrosted wild blueberries (*V. myrtillus* L.) were homogenized using Polytron (PT 1200E) (Kinematica, Luzern, Switzerland) for 5 min, then 5.000 g of the homogenized sample was extracted with 40 mL of acidified (0.5% HCl) aqueous ethanol solution (70% *v*/*v*). After a 24 h extraction at 25 °C with constant shaking at 160 rpm (Sklo Union LT, Teplice, Czech Republic), samples were filtered with a filter paper (Watman no.1) in a Buchner funnel and stored at 4 ± 1 °C until analyzed.

### 2.2. Analysis

#### 2.2.1. Determination of Total Phenolic Content (TPC)

The total phenolic contents of berry extracts were determined using the Folin–Ciocalteu method as previously described by Bobinaite et al. [32]. Briefly, the test tubes were filled with 1.0 mL of appropriately diluted extract and mixed with 5.0 mL of Folin–Ciocalteu’s phenol reagent diluted in distilled water (1/10, *v*/*v*) and 4.0 mL of Na_2_CO_3_ (7.5%). The absorbance of the test solution was read at 765 nm after 60 min of incubation in the darkness using a Genesys-10 UV/Vis spectrophotometer (Thermo Spectronic, Rochester, NY, USA). Gallic acid was used as the standard for the calibration curve and results are expressed in mg of gallic acid equivalents in 100 g of berries (FW) (Table 1).

#### 2.2.2. HPLC Analysis of Total Anthocyanin Content (TAC)

Anthocyanins were analysed using a Waters 2695 series HPLC system, equipped with a Waters 2998 photo diode array detector (DAD) (Waters Corporation, Milford, MA, USA). Analytical separation was carried out using a LiChroCART Purospher^®^ STAR RP-18 endcapped column (250 × 4.6 mm, 5 µm particle size) with a guard column, Purospher STAR RP 18e 4.0 × 4.0 mm 5 µm (Merck KgaA, Darmstadt, Germany), using a slightly modified procedure of Lätti et al. [27]. The temperature of the column oven was set at 25 °C. The mobile phase consisted of aqueous 10% formic acid (eluent A) and ACN–MeOH (85:15, *v*/*v*) (eluent B). The gradient program was as follows: 0–2 min, 4–6% eluent B; 2–4 min, 6–8% eluent B; 4–12 min, 8–9% eluent B; 12–46 min, 9–11% eluent B; 46–48 min, 11–24% eluent B; 48–52 min, 24–34% eluent B; 52–59 min, 34–80% eluent B; 59–61 min, 80–20% eluent B; 61–65 min, 4% eluent B. The injection volume was 10 µL.

Anthocyanins were detected at the wavelength of 520 nm. DAD data were recorded from 200 to 600 nm. Anthocyanins in bilberry extracts were identified according to the HPLC retention times (RT) and UV absorbance maximum, in comparison with commercial standards or with literature data [27]. The chromatogram is added to supplementary materials (Figure S1).

Commercial standard (cyanidin-3-glucoside) was dissolved in solvent B (10%) and solvent A (90%) to generate a seven-point external standard calibration curve (with a concentration range from 1 to 100 mg/L) whose linearity was acceptable (R^2^ = 0.999).

The total content of anthocyanins in the extracts was determined by the sum of the amounts of the individually quantified compounds as equivalents of cyanidin-3-glucoside (C_3_G) per 100 g of FW of berry (Table 1).

### 2.3. Determination of Ferric-Reducing Antioxidant Power (FRAP)

A FRAP assay was performed according to the method of Benzie and Strain [33], with slight modifications [4].

For the FRAP assay, 0.3 M of sodium acetate buffer (pH 3.6) was prepared by dissolving 3.1 g of sodium acetate and 16 mL of acetic acid in 1000 mL of distilled water; a 10 mM TPTZ solution was prepared by dissolving 0.031 g of TPTZ in 10 mL of 40 mM HCl; and a 20 mM ferric solution was prepared by dissolving 0.054 g of FeCl3·6H2O in 10 mL of distilled water. Working FRAP reagent was prepared by freshly mixing acetate buffer, TPTZ and ferric solutions at a ratio of 10:1:1. 

For the analysis, 2 mL of freshly prepared FRAP working solution and 20 µL of diluted extract were mixed and incubated for 30 min at ambient temperature. The change in absorbance due to the reduction of ferric-tripyridyltriazine (Fe III-TPTZ) complex by the antioxidants present in the samples was monitored at 593 nm using a Genesys-10 UV/Vis (Thermo Spectronic, Rochester, NY, USA) spectrophotometer. The absorptions of blank samples (by applying the same analysis conditions) were tested each time before and after analysis. 

Trolox was used as the standard and the antioxidant activity is expressed as µmol of trolox equivalents (µmol TE) per g of berries (FW) (Table 1).

### 2.4. Determination of ABTS Radical Scavenging Activity (ABTS RSA)

The RSA of extracts was also measured by ABTS^•+^ radical cation assay [34]. ABTS solution (2 mM) was prepared by dissolving 2,2′-azinobis(3-ethylbenzothiazoline-6-sulfonic acid) diammonium salt in 50 mL of phosphate-buffered saline (PBS) obtained by dissolving 8.18 g NaCl, 0.27 g KH_2_PO_4_, 1.42 g Na_2_HPO_4_ and 0.15 g KCl in 1 L of pure water. The pH of the prepared solution was adjusted to 7.4 using NaOH. Then K_2_S_2_O_8_ solution (70 mM) was prepared in pure water. 

The working solution (ABTS^•+^ radical cation) was produced by reacting 50 mL of ABTS solution with 200 μL of K_2_S_2_O_8_ solution and allowing the mixture to stand in the dark at room temperature for 15–16 h before use. 

For the assessment of antiradical activity of the extracts, 2 mL of ABTS^•+^ solution was mixed with 20 μL extract in a 1 cm path length cuvette. The reaction mixture was kept at room temperature in the dark for 30 min and the absorbance was read at 734 nm. 

Trolox was used as the standard and ABTS RSA is expressed as µmol of trolox equivalents (µmol TE) per g of berries (FW) (Table 1).

### 2.5. Determination of Dry Matter (DM), Total Soluble Solids (TSS) and pH

Dry matter content was determined after forced air convention drying at 105 °C to a constant weight (Table 1).

The total soluble solids were determined using a digital refractometer (ATAGO PR-32, Atago Co., Ltd., Tokyo, Japan) (Table 1).

The pH was measured using an inoLab Level 1 pH meter with a SenTix 81 (WTW) electrode (Table 1).

### 2.6. Statistical Analysis

All the experiments were carried out in triplicate. The mean values and standard deviations of the experimental data were calculated using SPSS 20 software (SPSS Inc., Chicago, IL, USA). One-way analysis of variance (ANOVA) along with the post hoc Tukey’s HSD test were employed for statistical analysis. Differences were considered to be significant at *p* < 0.05.

## 3. Results

### 3.1. Sample Locations

The ripe berries of bilberry (*V. myrtillus* L.) were handpicked during the summers of 2019 and 2020 during the time periods when they are typically harvested for commercial purposes in Norway, Finland, Latvia and Lithuania in three different locations (Table 2).

The berry samples were cooled immediately to below 10 °C then frozen and stored at −55 °C until use.

### 3.2. The pH and Total Soluble Solids (TSS) Content

**Figure 1:**
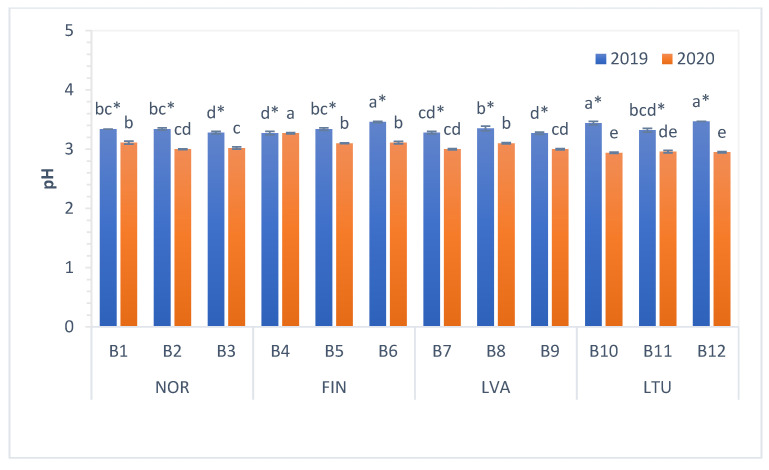
pH values of bilberries. Values are presented as means ± standard deviations. Different letters within the same column indicate significant differences between the collection locations (B1–B12) (*p* < 0.05). Significant differences between 2019 and 2020 are indicated by asterisks (*) (*p* < 0.05). Locations of bilberry sample collection include Norway (NOR), Finland (FIN), Latvia (LVA) and Lithuania (LTU).

The pH measures the acidity, and the total soluble solids (TSS) shows a high positive correlation with sugar content of fruits and berries [35]. The organoleptic quality and storage life of berries is related to their TSS content and acidity [36]. The pH values of investigated bilberries varied from 2.94 to 3.47 (Figure 1). In 2019, lower mean pH values were found in berries from LVA and NOR (3.30 and 3.32, respectively), whereas in 2020, the lowest mean pH was measured in bilberries from LTU (2.95). In 2020, the pH of bilberries from all countries was significantly lower than in 2019 (Figure 1). 

Previously, Giovanelli and Buratti (2009) [37] reported that the pH of Italian bilberries ranged from 3.13 to 3.22. Turkben et al. (2008) [38] reported pH values between 2.77 and 2.95 among wild bilberries from western Turkey. These results are in accordance with the pH values estimated in our study.

**Figure 2:**
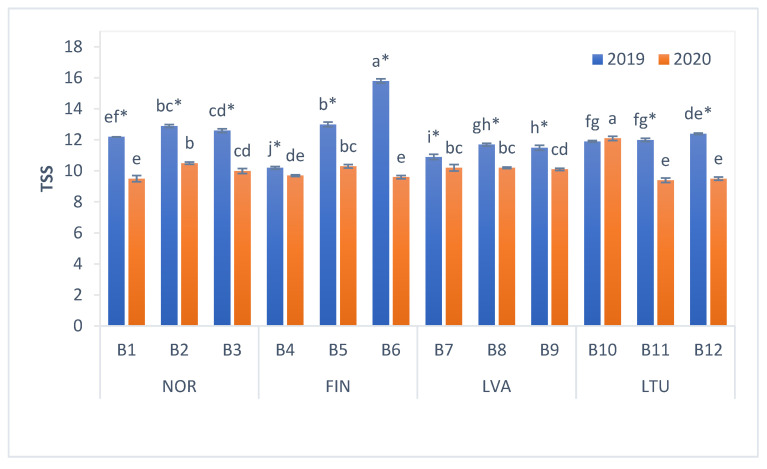
TSS values of bilberries. Values are presented as means ± standard deviations. Different letters within the same column indicate significant differences between the collection locations (B1–B12) (*p* < 0.05). Significant differences between 2019 and 2020 are indicated by asterisks (*) (*p* < 0.05). Locations of bilberry sample collection include Norway (NOR), Finland (FIN), Latvia (LVA) and Lithuania (LTU).

The content of TSS in bilberries varied from 9.4 to 15.8 Brix° (Figure 2). In 2019, berries from NOR and FIN had higher mean TSS content (12.6 and 13.0 Brix°, respectively) than berries from LVA and LTU, whereas in 2020, the mean TSS content of berries from all countries was similar (Figure 2). In 2020, TSS values of the bilberry samples, with the exception of the ones collected in LTU location B10, were significantly lower than the respective values determined in 2019. 

TSS values of investigated bilberries were in accordance with previously reported findings. Turkben et al. (2008) [38] reported that the TSS content in *V. myrtillus* berries from Turkey ranged from 9.0 to 11.0%. The TSS content in wild bilberries from Italy varied from 10.8 to 11.1% [37], whereas the TSS content in bilberries from Romania—from 9.2 to 13.7% [39]. TSS content in Polish bilberries was 13.0% [40]. Different growth and environment conditions such as temperature, day length and light intensity possibly influenced the TSS of the berries [15].

### 3.3. Total Phenolic Content (TPC)

**Figure 3:**
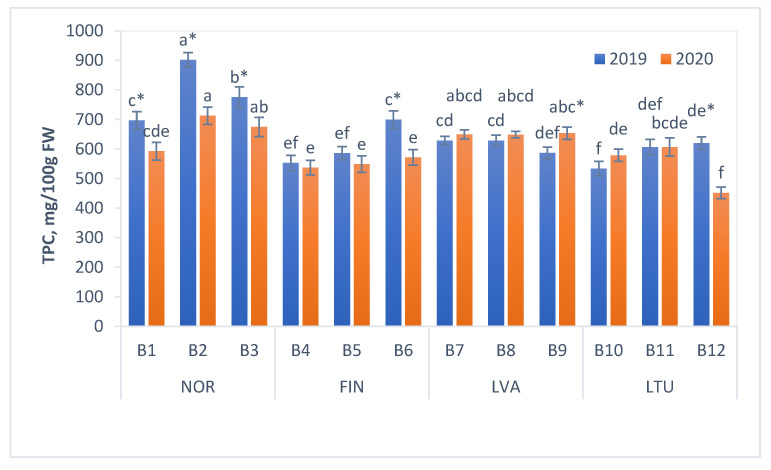
Total phenolic content (TPC) of bilberries mg/100 g FW. Values are presented as means ± standard deviations. Different letters within the same column indicate significant differences between the collection locations (B1–B12) (*p* < 0.05). Significant differences between 2019 and 2020 are indicated by asterisks (*) (*p* < 0.05). Locations of bilberry sample collection include Norway (NOR), Finland (FIN), Latvia (LVA) and Lithuania (LTU).

The total phenolic content (TPC) of the tested bilberry samples ranged from 452 (LTU B12 in 2020) to 902 mg/100 g FW (NOR B2 in 2019) (Figure 3). The highest average TPC value was found in the berries collected in Norway (791 mg/100 g FW in 2019 and 660 mg/100 g FW in 2020). For both years (in 2019 and 2020), the lowest mean TPC value was found in berry samples from Lithuania (587 mg/100 g FW in 2019 and 546 mg/100 g in 2020). 

Total phenolic contents of investigated samples were in accordance with previously reported findings. For instance, the TPC value of bilberries collected from a natural population in Macedonia was 706 mg/100 g FW [41] and in bilberries from Serbia—890 mg/100 g FW [42]. However, Milivojević et al. [43] determined a somewhat lower TPC of bilberries collected in Serbia (387 mg/100 g FW). The TPC of bilberries from the forest of Poland was reported to be 640 mg/100 g FW [39]. The TPC values of bilberries from natural populations in Norway were reported to range between 512 and 674 mg/100 g FW [44,45].

It has previously been shown that both the growing conditions and the genetic origin of the wild bilberries affect the contents of phenolic compounds [46,47]. The latitude-related factor was reported as having a high influence on the quality and quantity of phenolic compounds in bilberries, suggesting that higher phenolic contents may be supported by northern latitudes, altitude and sunny weather [48]. In previous studies, higher contents of phenolic compounds and anthocyanins were detected in bilberry clones originating from higher latitudes [27,46,49]. Interestingly, our data also show that the mean TPC of berries from Norway (the most northern country covered in the study) was the highest, whereas the mean TPC of samples from the most southern country (Lithuania) was the lowest (Figure 3). Furthermore, bilberry samples from Norway collected in the northernmost location (B2) had the highest TPC value, whereas the TPC values of the samples collected in the southernmost location (B1) in 2019 and 2020 were 23 and 17% lower, respectively. Similarly, among samples collected in Finland, the highest TPC value (700 mg/100 g FW in 2019 and 572 mg/100 g FW in 2020) was determined in berries from the northernmost location (B6) (Figure 3). In 2019, the same trend could also be observed for the bilberry samples from Lithuania, where TPC values also slightly increased with higher latitudes. On the other hand, for Lithuanian samples, this trend was not observed in 2020. The samples collected in Latvia had very similar TPC values, most likely due to the proximity of the sample collection sites. Our results also indicate that there were significant yearly variations in the TPC values of berries (Figure 3), suggesting that although genotype affects the TPC in bilberries, its final content also depends on weather conditions.

### 3.4. Total Anthocyanin Content (TAC)

**Figure 4:**
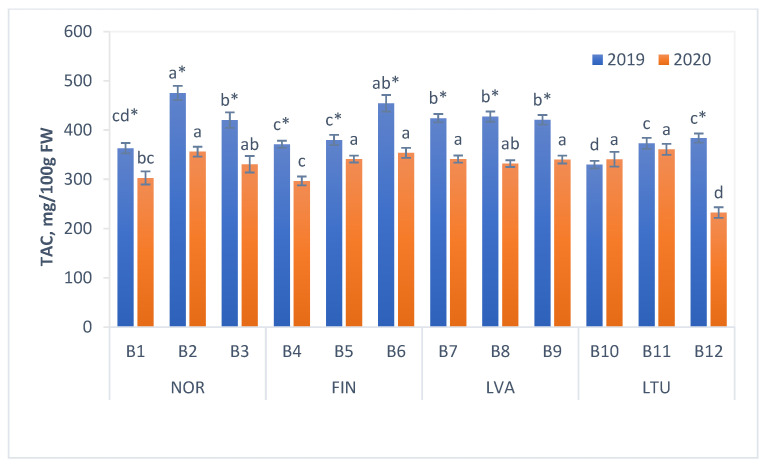
Total anthocyanin content (TAC) of bilberries mg/100 g FW. Values are presented as means ± standard deviations. Different letters within the same column indicate significant differences between the collection locations (B1–12) (*p* < 0.05). Significant differences between 2019 and 2020 are indicated by asterisks (*) (*p* < 0.05). Locations of bilberry samples collection include Norway (NOR), Finland (FIN), Latvia (LVA) and Lithuania (LTU).

Bilberry is one of the richest sources of anthocyanins, which have multiple biological activities [11]. The mean TAC values of investigated bilberry samples were 401.9 and 327.5 mg/100 g FW in 2019 and 2020, respectively. It is worth noting that within the same year, the mean TAC values of the berry samples from different countries did not differ significantly, with one exception—Lithuanian bilberries in 2019, which had a significantly lower mean TAC (Figure 4). The highest TAC values were from two berry samples collected in 2019 in the northernmost locations (B2 and B6) in Norway and Finland (475.4 and 454.6 mg/100 g FW, respectively). 

TAC values of investigated bilberry samples were in accordance with previously reported findings. For instance, Skrede and colleagues [44] reported that the concentration of anthocyanins in bilberry samples ranged from 429 to 627 mg/100 g FW [44]. The TAC of bilberries from Macedonia was 507 mg/100 g FW [41]. Rohloff et al. [45] reported somewhat lower amounts of total anthocyanins in Norwegian bilberries (from 330 to 449 mg/100 g FW) [45], whereas the TAC of Finnish bilberries from 20 different populations varied from 350 to 525 mg/100 g FW [27].

It has been shown that genotype and environment interaction affect accumulation of anthocyanins in bilberries [7,45,47]. The increasing trend in anthocyanin content has been repeatedly observed in bilberries toward northern latitudes of Europe [27,49]. However, when effects of different environmental factors on berry chemical composition were studied in eight forest fields of bilberry in the north, the middle and the south of Norway, previous findings concerning latitudinal effects on anthocyanin concentration were not confirmed [45]. The authors concluded that environmental impacts probably confounded the genetic (population) effects [45]. 

With regard to increased TAC towards northern latitudes, the trend was not clear in the present study. In 2019, the highest TAC values were from two samples collected in the northernmost locations (B2 and B6), however, in 2020, the berries from locations B2 and B6 had high, but not the highest TAC values (Figure 4).

In 2020, TAC of the bilberry samples, with the exception of the ones collected in LTU location B10, were 3 to 39% lower than the respective values determined in 2019, which suggest significant influence of environmental factors on the accumulation of anthocyanins.

### 3.5. Antioxidant Activity (AA)

The antioxidant activity of berry samples was evaluated using FRAP and ABTS assays. In the ABTS assay, antioxidants suppress the generation of a blue-green ABTS radical cation by electron donation radical scavenging, whereas in the FRAP assay, there are no free radicals involved, but the reduction of ferric-to-ferrous iron is monitored. 

**Figure 5:**
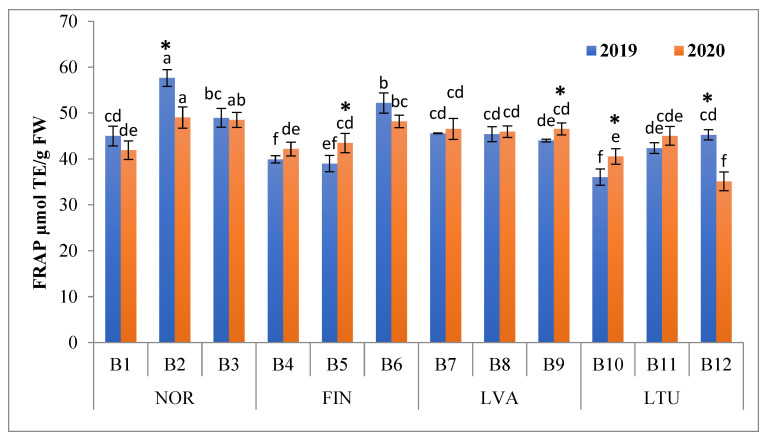
Ferric-reducing antioxidant power (FRAP) of bilberries (μmol TE/g FW). Different letters above the same color bars indicate significant differences between the mean values (*p* < 0.05). Significant differences between 2019 and 2020 are indicated by asterisks (*) (*p* < 0.05). Locations of bilberry samples collection include Norway (NOR), Finland (FIN), Latvia (LVA) and Lithuania (LTU).

In 2019, the FRAP of bilberry samples ranged from 36.0 (LTU B10) to 57.7 μmol TE/g FW (NOR B2) and in 2020, from 35.1 (LTU B12) to 49.1 μmol TE/g FW (NOR B2) (Figure 5). For both years of investigation, the highest mean FRAP values were from berry samples collected in Norway (50.6 μmol TE/g and 46.6 μmol TE/g FW in 2019 and 2020, respectively), followed by samples collected in Latvia (45.0 μmol TE/g and 46.3 μmol TE/g FW in 2019 and 2020, respectively). Bilberry samples from Lithuania had the lowest mean FRAP values (41.2 μmol TE/g and 40.2 μmol TE/g FW in 2019 and 2020, respectively). The FRAP results obtained in our study are close to those previously reported (53 and 57 μmol TE/g FW) in *V. myrtillus* fruits [50].

**Figure 6:**
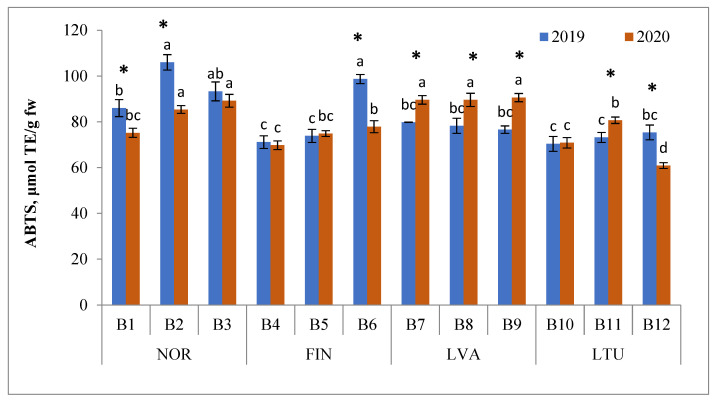
ABTS radical scavenging activity (RSA) of bilberries (μmol TE/g FW). Different letters above the same color bars indicate significant differences between the mean values (*p* < 0.05). Significant differences between 2019 and 2020 are indicated by asterisks (*) (*p* < 0.05). Locations of bilberry samples collection include Norway (NOR), Finland (FIN), Latvia (LVA) and Lithuania (LTU).

Berries showed higher antioxidant activity in the ABTS reaction system (Figure 6). The ABTS RSA of bilberries ranged from 60.9 (LTU B12 in 2020) to 106.0 μmol TE/g FW (NOR B2 in 2020). In 2019, the highest mean ABTS RSA was from berry samples collected in Norway (95.1 μmol TE/g FW), followed by samples collected in Finland (81.3 μmol TE/g FW), whereas in 2020, the highest mean ABTS RSA was from berry samples from Latvia (89.9 μmol TE/g FW) followed by samples from Norway (83.3 μmol TE/g FW) (Figure 6).

It was observed that bilberries from different geographical locations vary significantly in quantity of antioxidant activity. It is stated that plants are exposed to natural climatic stress due to environmental differences. This study’s results show that in the case of wild bilberries, the natural variation in biologically active compounds and antioxidant activity has shown an authenticity of the berries from northern countries in comparison to more southern natural habits.

In agreement with previous findings [37,51] a high positive correlation was found between TPC and antioxidant activity of the bilberry samples (R = 0.88 and 0.91 as determined by the FRAP and ABTS assays, respectively), whereas the correlation between TAC and antioxidant activity was somewhat lower (R = 0.65 and 0.60 as determined by the FRAP and ABTS assays, respectively).

Previously, Giovanelli and Buratti [37] investigated cultivated blueberries (*Vaccinium corymbosum*) and wild bilberries (*Vaccinium myrtillus*) and reported that the antioxidant capacity of berries strongly correlated with the content of total anthocyanins and total phenolics. However, contrary to our findings, a higher correlation coefficient was found between the antioxidant capacity and total anthocyanin content (R = 0.93) than between the antioxidant capacity and total phenolic content (R = 0.89) of berries [37]. Uleberg et al. [46] also reported quite strong correlations between anthocyanins, total phenolics and antioxidant activity of bilberries.

## 4. Conclusions

With the increasing globalization of the food supply chain, food quality and safety have become of increased concern for consumers, food producers and governments. Although there is no reconciled definition of food fraud, it is generally accepted that food fraud is committed intentionally for financial gain through consumers. Nowadays, the most common food categories susceptible to any type of food fraud are the so-called premium food products, including non-timber forest products such as bilberries. The biochemical composition and nutritional value of berries are particularly relevant in the production of various pharmaceutical products and food supplements. In addition, there are natural or unavoidable flaws which may be caused by climatic or annual conditions. Nevertheless, berry authenticity research is important to ensure that the berries offered for sale are of the nature, substance and quality expected by the purchaser. Bilberries have different amounts of bioactive substances (anthocyanins, phenolics, etc.), depending on the place of growth and climatic conditions, so buyers are interested in knowing where those berries came from, as berries from certain regions or countries will also fetch a higher price due to their unique qualities. Spectrophotometric methods are fast and easy and therefore highly suitable to be used in the comparison of berry quality, especially for commercial purposes. 

This study provided information concerning polyphenol (TPC) and total anthocyanin (TAC) content, antioxidant capacity (FRAP; ABTS RSA) and physico-chemical properties (total soluble solids (TSS), pH) of wild bilberry (*V. myrtillus* L.) populations from the Northern Europe (Norway (NOR), Finland (FIN), Latvia (LVA) and Lithuania (LTU)), including three locations in each country. Variations in biologically active compounds, antioxidant activity and physico-chemical properties in wild bilberries (*Vaccinium myrtillus* L.) have not been previously systematically investigated. Some studies have been conducted in order to evaluate the extent of the variation in the anthocyanins of bilberries or blueberries in one country, but there is little information in the results indicating geomorphological influence on the biochemical and physico-chemical compositions of bilberries depending on their place of origin. These may be used as discriminating criteria for distinguishing between bilberries from different geographical origins.

In conclusion, our study results show that the pH values of investigated bilberries varied from 2.94 to 3.47. These results are in accordance with the pH values estimated from literature data (2.77–3.22) [36,37]. The TSS content of bilberries varied within a similar range—from 9.4 to 15.8 Brix°. In 2020, the pH values and TSS content of berries were significantly lower than in 2019, showing that different growth and environment conditions such as temperature, day length and light intensity possibly influence the TSS of the berries.

The TPC of bilberries ranged from 452 to 902 mg/100 g FW. The mean TPC of bilberries from Norway (the most northern country covered in the study) was the highest, whereas the mean TPC of samples from the most southern country (Lithuania) was the lowest. The TAC values of investigated bilberry samples varied from 232.7 to 475.5 mg/100 g FW and were somewhat lower in 2020 than in 2019, which could be due to several factors, including genetic variation and environmental conditions.

The ABTS RSA of bilberries ranged from 60.9 (LTU B12 in 2020) to 106.0 μmol TE/g FW (NOR B2 in 2020). In 2019, the highest mean ABTS RSA had berry samples collected in Norway (95.1 μmol TE/g FW), followed by samples collected in Finland (81.3 μmol TE/g FW), whereas in 2020, the highest mean ABTS RSA was from berry samples from Latvia (89.9 μmol TE/g FW) followed by samples from Norway (83.3 μmol TE/g FW). A high positive correlation was found between TPC and antioxidant activity of the bilberry samples (R = 0.88 and 0.91 as determined by the FRAP and ABTS assays, respectively), whereas the correlation between TAC and antioxidant activity was lower (R = 0.65 and 0.60 as determined by the FRAP and ABTS assays, respectively).

In recent years, a growing demand for healthy food has been noted in the market. Consumers are primarily interested in food which is appealing, helps in preventing various diseases and contains high levels of promoted bioactive compounds. Our results could therefore be very useful for nutritional studies on anthocyanin- and phenolic compound-rich food and berry authenticity research. Results indicated a geomorphological influence on the biochemical and physico-chemical compositions of bilberries depending on their place of origin. These may be used as discriminating criteria for distinguishing between bilberries from different geographical origins.

## Figures and Tables

**Table 1:** Summary of analysis methods.

Analysis Method	Assay	Unit
Polyphenol content (TPC)	Folin–Ciocalteu	mg GAE/100 g FW
Total anthocyanin content (TAC)	Liquid chromatography	mg C_3_G/100 g FW
Antioxidant capacity	FRAP, ABTS	μmol TE/g FW
Total soluble solids (TSS)	Refractometric	Brix°
pH	Potentiometric	-

**Table 2:** Locations of bilberry sample collection in Norway (NOR), Finland (FIN), Latvia (LVA) and Lithuania (LTU).

Sample No. (Collection Location)	Country Code	Coordinates	
		Latitude	Longitude
B1	NOR	69°41.66521′	18°59.46854′
B2	NOR	69°45.07693′	19°01.54336′
B3	NOR	69°40.25058′	18°37.08972′
B4	FIN	64°51.69040′	26°42.26600′
B5	FIN	64°59.17020′	25°54.21950′
B6	FIN	65°13.75280′	25°33.59240′
B7	LVA	57°08.55168′	21°51.95172′
B8	LVA	57°09.05976′	21°51.08952′
B9	LVA	57°08.79108′	21°52.32660′
B10	LTU	54°07.37150′	24°43.01752′
B11	LTU	54°43.29541′	23°30.53200′
B12	LTU	55°04.48091′	22°28.23829′

## Data Availability

Data is contained within the article and Supplementary Materials.

## Supplementary Materials

The following supporting information can be downloaded online: https://www.mdpi.com/article/10.3390/antiox11030588/s1, Figure S1: HPLC-PDA chromatogram (λ = 520 nm) of the wild bilberry B2 (*Vaccinium myrtillus* L.) samples extracts of 2019 and 2020.
