# Supplementary material for: Geographic Variability of Biologically Active Compounds, Antioxidant Activity and Physico-Chemical Properties in Wild Bilberries (Vaccinium myrtillus L.)
Source: Antioxidants (Basel). 2022 Mar 19;11(3):588. doi: 10.3390/antiox11030588 (PMC8945452; doi:10.3390/antiox11030588)
Supplement: Supplementary file 1 [file antioxidants-11-00588-s001.zip › antioxidants-1588669-supplementary.pdf]

Supplementary material:

Geographic variability of biologically active compounds, antioxidant activity and physico-chemical properties in wild bilberries (*Vaccinium myrtillus* L.)

Dalia Urbonaviciene [1,*], Ramune Bobinaite [1], Pranas Viskelis [1], Ceslovas Bobinas [1], Aistis Petruskevicius [1], Linards Klavins[2] and Jonas Viskelis [1]

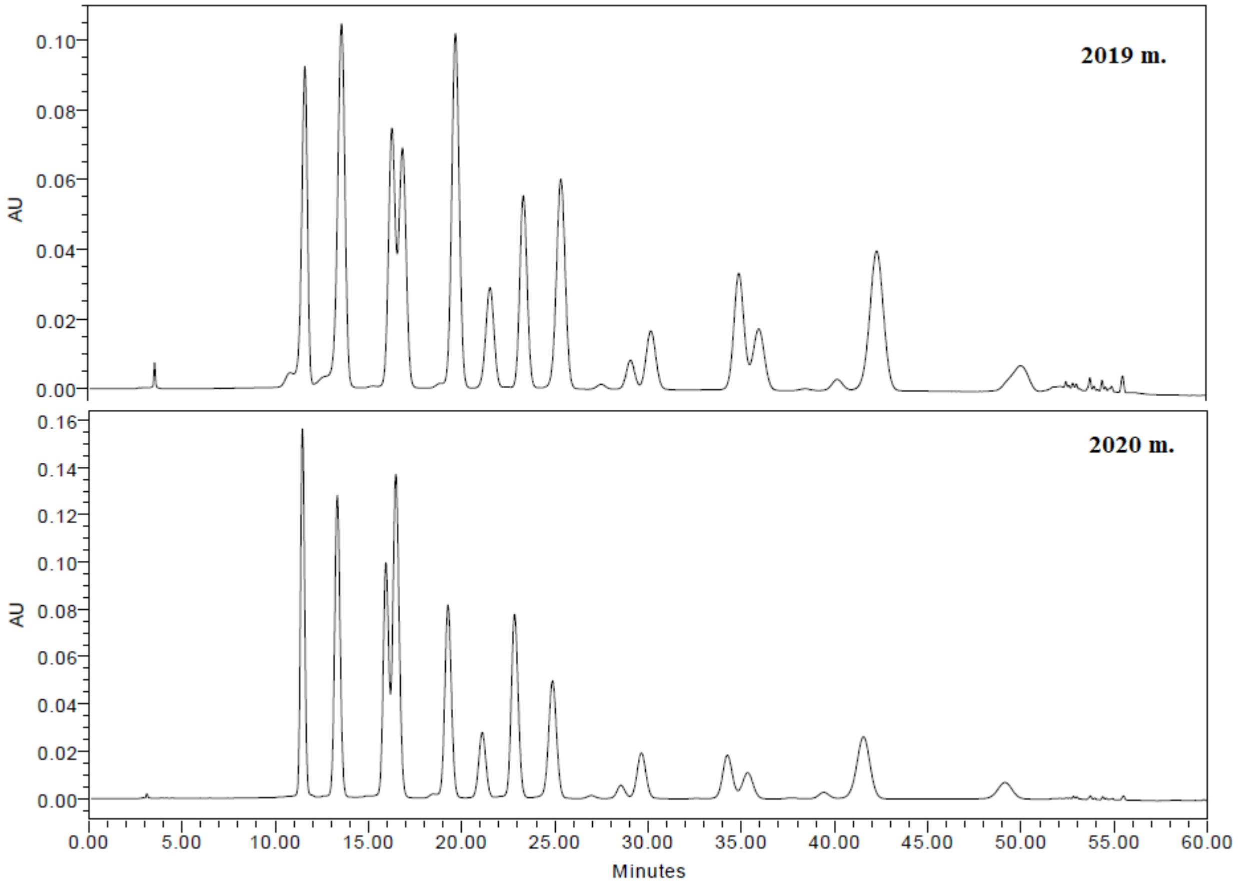

Figure S1. HPLC-PDA chromatogram (λ = 520 nm) of the wild bilberry B2 (*Vaccinium myrtillus* L.) samples extracts of 2019 and 2020.
